# Cortisol Levels in Childhood Associated With Emergence of Attenuated Psychotic Symptoms in Early Adulthood

**DOI:** 10.1016/j.biopsych.2021.08.009

**Published:** 2021-08-19

**Authors:** Alexis E. Cullen, Helen L. Fisher, Nancy Gullet, Elizabeth R. Fraser, Ruth E. Roberts, Uzma Zahid, Melody To, Natalie Huijing Yap, Patricia A. Zunszain, Carmine M. Pariante, Stephen J. Wood, Philip McGuire, Robin M. Murray, Valeria Mondelli, Kristin R. Laurens

## Abstract

**Background:**

In individuals at clinical high-risk for psychosis, elevated cortisol levels predict subsequent onset of psychotic disorder. However, it is unclear whether cortisol alterations are evident at an earlier clinical stage and promote progression of psychosis expression. This study aimed to address this issue by investigating whether cortisol levels in childhood were associated with the emergence of attenuated psychotic symptoms in early adulthood. In exploratory analyses, we examined whether cortisol and psychosocial stress measures interacted in predicting attenuated psychotic symptoms.

**Methods:**

A sample of children (*N* = 109) enriched for psychosis risk factors were recruited at age 9–12 years and assessed at age 11–14 years (T1) and 17–21 years (T2). Measures of psychopathology, psychosocial stressors, and salivary cortisol were obtained at T1. Attenuated psychotic symptoms were assessed at T2 using the Prodromal Questionnaire.

**Results:**

Diurnal cortisol (β = 0.915, 95% CI: 0.062–1.769) and daily stressors (β = 0.379, 95% CI: 0.034–0.723) at T1 were independently associated with total Prodromal Questionnaire scores at T2 after accounting for demographic factors and T1 psychopathology. Exploratory analyses indicated a significant interaction between T1 diurnal cortisol and daily stressors (β = 0.743, 95% CI: 0.081–1.405), with the highest predicted T2 total Prodromal Questionnaire scores occurring when both diurnal cortisol and daily stressors were increased.

**Conclusions:**

Our findings suggest that daily stressors and elevations in diurnal cortisol in late childhood/early adolescence increases risk for developing attenuated psychotic symptoms. These findings emphasize the importance of assessing environmental and biological risk factors for psychosis during neurodevelopmentally vulnerable time periods.

There is now convincing evidence implicating psychosocial stressors (e.g., major life events, daily stressors, and childhood trauma) in the development and exacerbation of psychotic disorders (1–7). While the biological processes underlying these findings have yet to be fully elucidated, the neural diathesis-stress model proposes that individuals with increased vulnerability for psychosis have abnormalities within the hypothalamic-pituitary-adrenal (HPA) axis that render them more sensitive to the effects of psychosocial stressors (8–10), and that these abnormalities contribute to the dopaminergic and glutamatergic abnormalities that underlie psychotic symptoms (11). Supporting this, individuals with psychosis and those at risk for the disorder (due to a family history of psychosis and/or clinical features) have been found to show HPA axis alterations, although meta-analyses indicate substantial heterogeneity across studies (12–16). These alterations include elevations in basal and diurnal cortisol levels in blood and saliva^1^ (12–14,17–21) and increased pituitary volume (15,21,22) but diminished salivary cortisol secretion in response to acute stress exposure (16,23–25) and awakening^2^ (21,26–28).

While cross-sectional studies of at-risk individuals have shown that these groups have similar cortisol profiles to those observed in patients with established psychosis (18–20,24,25,27–30), no clear pattern has yet emerged in longitudinal studies examining the relationship between baseline cortisol and transition to full psychosis. Such longitudinal studies have focused largely on help-seeking individuals who present with a clinical high-risk (CHR) state, typically featuring attenuated psychotic (AP) symptoms (31). Small studies (*N* < 40) comparing baseline cortisol levels in CHR individuals who later transitioned to psychosis and those who did not have produced mixed findings for basal plasma/ serum cortisol (32,33) and salivary cortisol awakening response (CAR) (33,34) measures. Concerning basal salivary cortisol, a meta-analysis of 4 studies of CHR individuals (with sample sizes ranging from 33 to 256) found no association with transition status (13). However, a subsequent publication from the North American Prodrome Longitudinal Study (NAPLS-2), with a larger sample (*N* = 417) than previous studies, found that higher basal salivary cortisol at baseline predicted psychosis transition in univariable analyses and significantly increased the predictive ability of multivariable models, which included baseline positive symptoms and functioning (35). However, studies have yet to examine the association between diurnal cortisol [which may provide a more nuanced measure of HPA axis function (36)] and transition in at-risk individuals.

Findings from longitudinal studies examining psychosocial stressors have been equally variable. In a small study of CHR individuals (*N* = 39), those who transitioned to psychosis within 1 year reported greater exposure to stressful life events and higher levels of perceived stress at baseline than those who did not (33). Similarly, an early report from the NAPLS-2 cohort (*N* = 314) indicated that CHR individuals who transitioned within 2 years experienced greater life event exposure and distress at baseline (37). In contrast, 2 studies, one utilizing data from the NAPLS-2 cohort [*N* = 596 (38)] and the other a sample of Australian CHR individuals [*N* = 74 (39)], found that neither stressful life events nor childhood trauma were independently associated with transition at 1–2 years in multivariable analyses, and that they made little contribution to the predictive models. These findings imply that psychosocial stressors may have limited ability to predict transition in CHR individuals over and above baseline symptoms and demographic factors.

The strong associations of symptom severity and functioning at baseline with later transition to psychosis among CHR individuals (40) may contribute to the inconsistent findings from studies employing univariable versus multivariable analyses to examine associations between stress/cortisol and transition status. Alternatively, given that transition rates in CHR individuals are relatively low [most recently estimated at 22% over 3 years (40)], smaller studies focusing on this outcome may have been underpowered to detect an effect of these variables. Investigating the effects of cortisol and psychosocial stress on the development of AP symptoms (particularly when measured on a continuous scale), rather than transition to full psychosis, may therefore provide greater insights into the etiological role of these factors. To this end, we used data from an established longitudinal cohort of children (41), enriched for risk factors for schizophrenia, to determine whether salivary cortisol and psychosocial stressors at age 11–14 years were independently associated with development of AP symptoms at age 17–21, after accounting for prior psychopathology and demographic factors. Because we were motivated to investigate the mechanisms proposed by the neural diathesis-stress model (i.e., that subtle alterations in HPA axis function might render individuals more susceptible to the effects of stress), we further explored interactions between cortisol and stress in predicting AP symptoms.

## Methods and Materials

### Participants and Procedure

Participants were drawn from an established longitudinal investigation of children recruited from Greater London, United Kingdom at age 9–12 years [(41–43); see Supplement]. Children were identified using a school-screening procedure assessing well-established risk factors for schizophrenia (speech/motor developmental delays, internalizing/externalizing psychopathology, psychotic-like experiences, and a family history of psychosis) supplemented by targeted recruitment of child relatives of patients with schizophrenia/ schizoaffective disorder. The cohort was enriched with children presenting with one or more risk factors for schizophrenia but also included children with no reported risk factors.

Participants completed a range of biological, psychosocial, and cognitive assessments at approximately 2-year intervals throughout adolescence. At the initial assessment (when participants were aged 9–12 years), caregivers completed the Family Interview for Genetic Studies (44); this information was used to confirm a family history of schizophrenia/schizo-affective disorder (i.e., at least one first- or second-degree relative with a confirmed diagnosis; coded no vs. yes) and determine participant race (recategorized as African/Carib-bean, Black, Other, and White). This study examined data obtained during follow-up assessments completed at ages 11–14 years (psychopathology, salivary cortisol, and psychosocial stressors) and 17–21 years (AP symptoms), herein referred to as time 1 (T1) and time 2 (T2), respectively. At each assessment, children and their caregivers provided written informed assent and consent, respectively, to participate; on account of their age (>16 years), participants typically attended the T2 assessments unaccompanied by their caregivers and provided written consent to participate. Ethical approvals were granted by the Joint South London and Maudsley and the Institute of Psychiatry NHS Research Ethics Committee and the King’s College London, Psychiatry, Nursing and Midwifery Research Ethics Committee.

### Psychopathology

At the T1 assessment, participants completed the Youth Self-Report (45), an extensively used measure of childhood psychopathology that exhibits high reliability and validity. This 112-item questionnaire assesses problems occurring during the past 6 months on a 3-point scale (0, not true; 1, somewhat true or sometimes true; or 2, very true or often true), and provides overall age- and sex-normed T scores indexing internalizing (anxious/depressed, withdrawn/ depressed, somatic complaints) and externalizing (rule-breaking behavior, aggressive behavior) problems. Participants additionally completed the 9-item Psychotic-Like Experiences Questionnaire for Children (42,46) assessing a range of hallucination- and delusion-like experiences. This measure has established construct (46) and criterion validity (47). The 9 items are rated on a 3-point scale (0, not true; 1, somewhat true; or 2, certainly true) with items summed to derive a total score (maximum 18). To obtain a single variable, we performed principal component analysis on the Youth Self-Report internalizing, Youth Self-Report externalizing, and total Psychotic-Like Experiences Questionnaire for Children scores (see Supplement) and retained scores for the first principal component for use in all analyses (herein referred to as T1 psychopathology).

### Salivary Cortisol

Participants completed an established saliva sample collection protocol at home (27,28,48) within 1 month of the T1 assessment visit. Verbal and written instructions were provided for collecting saliva samples using the passive drool procedure (http://www.salimetrics.com). Participants collected six saliva samples throughout the day on 2 consecutive days (awakening; 15, 30, and 60 min after awakening; 12:00 noon; and 8:00 PM) and were instructed to wake before 10:00 AM, collect the first sample immediately on awakening, avoid food consumption for 30 minutes before each sample collection, and refrain from strenuous exercise (28). Protocol compliance was assessed using a self-report sampling diary, completed after each sample. Body mass index (BMI; kg/m^2^) was computed from measurements of participant height and weight obtained at the T1 assessment.

The cortisol assay procedure has been described previously (49). In brief, samples were stored in the participant’s home freezer until collection by a member of the research team and subsequently frozen at −20 °C at the laboratory. After thawing and centrifugation at 3000 rpm for 15 minutes, cortisol levels were determined using the Salimetrics High Sensitivity Salivary Cortisol ELISA KIT (Salimetrics), according to the recommended procedure. The analytic sensitivity was set to 0.33 nmol/L. Inter- and intra-assay coefficients (range: 8%−11% and 6%−10%, respectively) were within the acceptable ranges reported by the assay manufacturers. Consistent with previous publications using the saliva home-collection protocol (27,28,48,50), summary values indexing the CAR and diurnal cortisol (overall output) were derived using area under the curve computations (51). The CAR was calculated with respect to the increase in cortisol levels following awakening (using the awakening, 15, 30, and 60-min after awakening values), and diurnal cortisol was calculated with respect to ground for cortisol levels during the day (using awakening, 12:00 noon, and 8:00 PM values). Cortisol values for each time point were significantly correlated across testing days (*r_s_* range: 0.284–0.525, all *p* < .001). Because participants were typically more compliant with the sampling protocol on the first sampling day (i.e., fewer missing samples and better adherence to waking time and exercise restrictions), these data were used for area under the curve computations except when better compliance was demonstrated on the second day.

### Psychosocial Stress

At the T1 visit, participants completed 2 self-report psychosocial stress measures assessing daily stressors and negative life events (52). The daily stressor measure captured frequency of exposure (range: 0, never to 3, often) to 37 school-related events pertaining to schoolwork, peers, teachers, and home during the past 6 months and the degree of distress (range: 0, not at all to 3, a lot) experienced in relationship to each event (53). The negative life event measure assessed lifetime exposure (no vs. yes) to 8 child-appropriate events (53), such as parental separation/divorce, death of someone close, and serious illness, and the degree of distress experienced in relationship to each event (range: 0, not at all to 3, a lot). For each measure (daily stressors, negative life events), ratings were summed across items to derive a total exposure score and a total distress score; principal component analysis was applied to the two sets of total scores (with scores on the first component retained) to derive an overall daily stressor score and overall negative life event score (see Supplement for full details).

### AP Symptoms

AP symptoms were assessed at the T2 visit using the Prodromal Questionnaire (PQ) (54). The PQ is among the most widely used CHR screening tools (55) and has been evaluated in adolescents and adults (aged 12–35 years) recruited from general mental health settings and specialized prodromal research clinics (54,56,57). This self-report measure is composed of 92 items (rated true or false) assessing positive (45 items), negative (19 items), disorganized (13 items), and general (15 items) symptoms. The PQ therefore captures a broader range of AP symptoms than the Psychotic-Like Experiences Questionnaire for Children (which includes positive symptoms only). Items can be summed to derive a total PQ score, which demonstrates high internal consistency and is strongly associated with fulfillment of CHR criteria as assessed using structured clinical interviews (54).

### Data Analyses

Analyses were performed using Stata (version 16; StataCorp LLC); statistical significance was set at *p* < .05 for all tests. Ladder and gladder commands were used to assess the distribution of continuous variables and identify transformations to improve distributions where necessary; BMI (1/square), diurnal cortisol (log), and PQ total and positive scale scores (square root) were transformed accordingly.Variables that could not be improved to normal distribution by any transformation (age at T2, CAR, T1 psychopathology, negative life events) were retained in their original form and analyzed using nonparametric statistics. To identify covariates for inclusion in primary analyses, associations of demographic variables and T1 psychopathology with T1 predictor variables and T2 total and positive scale PQ scores were examined using Pearson’s product moment (*r*) and Spearman’s (*r_s_*) correlations (for continuous-continuous and binary-continuous pairings) and Kruskal-Wallis tests (for categorical-continuous pairings).

A multivariable linear regression model was used to test the effect of T1 predictor variables on T2 total PQ score (model specification provided in Supplement). We first derived the base model for total PQ scores, which included T1 psychopathology and demographic factors found to be associated with any T1 predictor variable or T2 total PQ scores. We then sequentially added predictor variables in blocks (cortisol variables only, stress variables only, cortisol and stress variables) to the base model and examined the change in model performance (*R^2^*, adjusted *R^2^*, and *F* statistic values). All inferences regarding associations between predictors and outcome were determined from the full (final) model. Multicollinearity and normality of residuals were assessed for the full model, which confirmed that all assumptions necessary for linear regression analyses were met. Added-variable plots (scatterplots showing the relationship between each T1 predictor and T2 total PQ scores, adjusted for all covariates in the full model) with 95% CIs were derived using the Stata avciplot package (58).

In exploratory analyses, separate linear regression models were used to test statistical interactions between each T1 cortisol variable and each T1 stressor variable (cortisol × stress) on T2 total PQ scores. Owing to the small sample size, the four interaction effects were tested in separate regression analyses, where each interaction term was added to the base model described above (see Supplement). Significant interactions were visualized using contour plots.

Because the PQ positive symptom scale has been found to show the strongest association with CHR status (54), we repeated the above steps with T2 positive scale PQ score as the outcome variable in sensitivity analyses. We additionally repeated the analyses for both outcome variables (total PQ scores and positive scale PQ scores) using robust regression (where cases with larger residuals are assigned smaller weights) to minimize the influence of potential outliers.

## Results

### Sample Characteristics

This sample includes 109 participants who were assessed at T1 and T2 (participants included in the study and those lost to follow-up did not differ on demographic or predictor variables) (Table S10). The mean age (± SE) of this sample at T1 and T2 was 13.21 (± 0.11) and 17.66 (± 0.08) years, respectively (Table 1); 50 (45.9%) participants were male, with the majority of White race (56.0%). One-fifth (21.1%) of the sample had a family history of schizophrenia/ schizoaffective disorder.

### Identification of Covariates

As shown in Table 2, male sex was negatively associated with T1 CAR and T2 total PQ scores, while age at T2 was positively associated with daily stressors at T1. Consistent with previous reports (28,52), a family history of schizophrenia/schizo-affective disorder was associated negatively with T1 CAR and positively with negative life events. T1 psychopathology was positively correlated with T1 daily stressors and negative life events, and with T2 total and positive scale PQ scores. Kruskal-Wallis tests (data not shown) indicated that T2 total PQ scores (Kruskal-Wallis *H*_2_ = 8.07, *p* = .017) and positive PQ scores (Kruskal-Wallis *H*_2_ = 8.88, p = .011) differed across racial groups. Cortisol and psychosocial stress measures were not significantly correlated (Table S11), which was expected given that they were not collected on the same day (59,60), as discussed in Supplement.

### Effects of Cortisol and Psychosocial Stress on AP Symptoms

**Figure 1:**
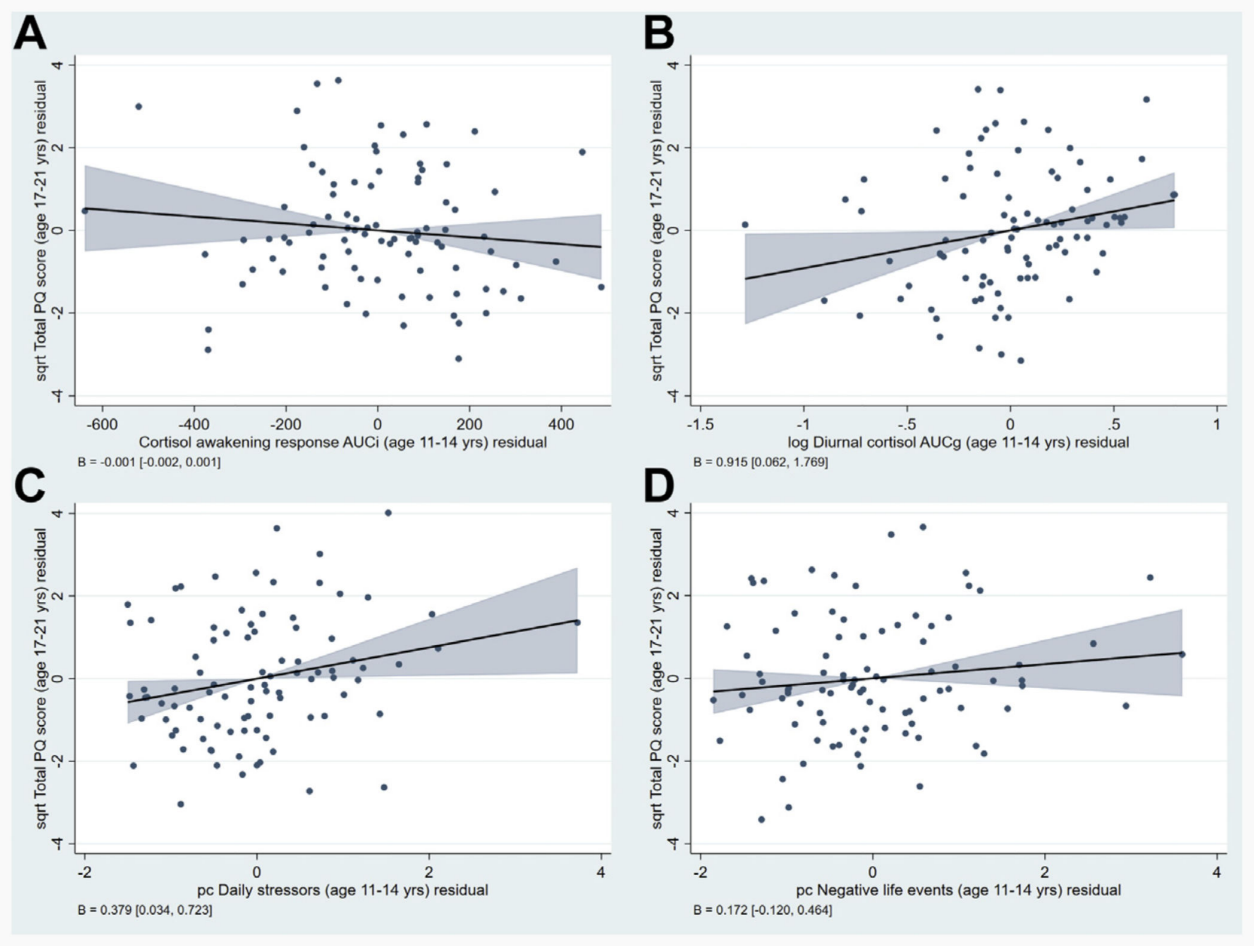
Associations between cortisol measures and psychosocial stressors at time 1 (age 11–14 years) and total Prodromal Questionnaire (PQ) score at time 2 (age 17–21 years). Added-variable plots showing relationships between the cortisol awakening response **(A)**, diurnal cortisol **(B)**, daily stressors **(C)**, and negative life events **(D)** at time 1 and total PQ scores at time 2 as derived from the full model (Table 3). AUCg, area under the curve with respect to ground; AUCi, area under the curve with respect to increase; pc, principal component (score for first pc); sqrt, square root transformed.

The proportion of variance explained in total PQ score after accounting for the number of predictors (adjusted *R*^2^) increased by 5% when either cortisol or stress variables were added to the base model (which included sex, age at T2, race, family history of schizophrenia/schizoaffective disorder, and T1 psychopathology) and by a further 5% when both were added simultaneously (Table 3). In the final (full) model (Table 4), T1 diurnal cortisol (β = 0.915, 95% CI: 0.062–1.769), T1 daily stressors (β = 0.379, 95% CI: 0.034–0.723), and male sex (β = −0.872, 95% CI: –1.555 to –0.188) were significantly associated with total PQ score at T2. Associations of cortisol and stress variables with total PQ score in the full model are illustrated in Figure 1.

### Exploratory Analyses Testing Interaction Effects

**Figure 2:**
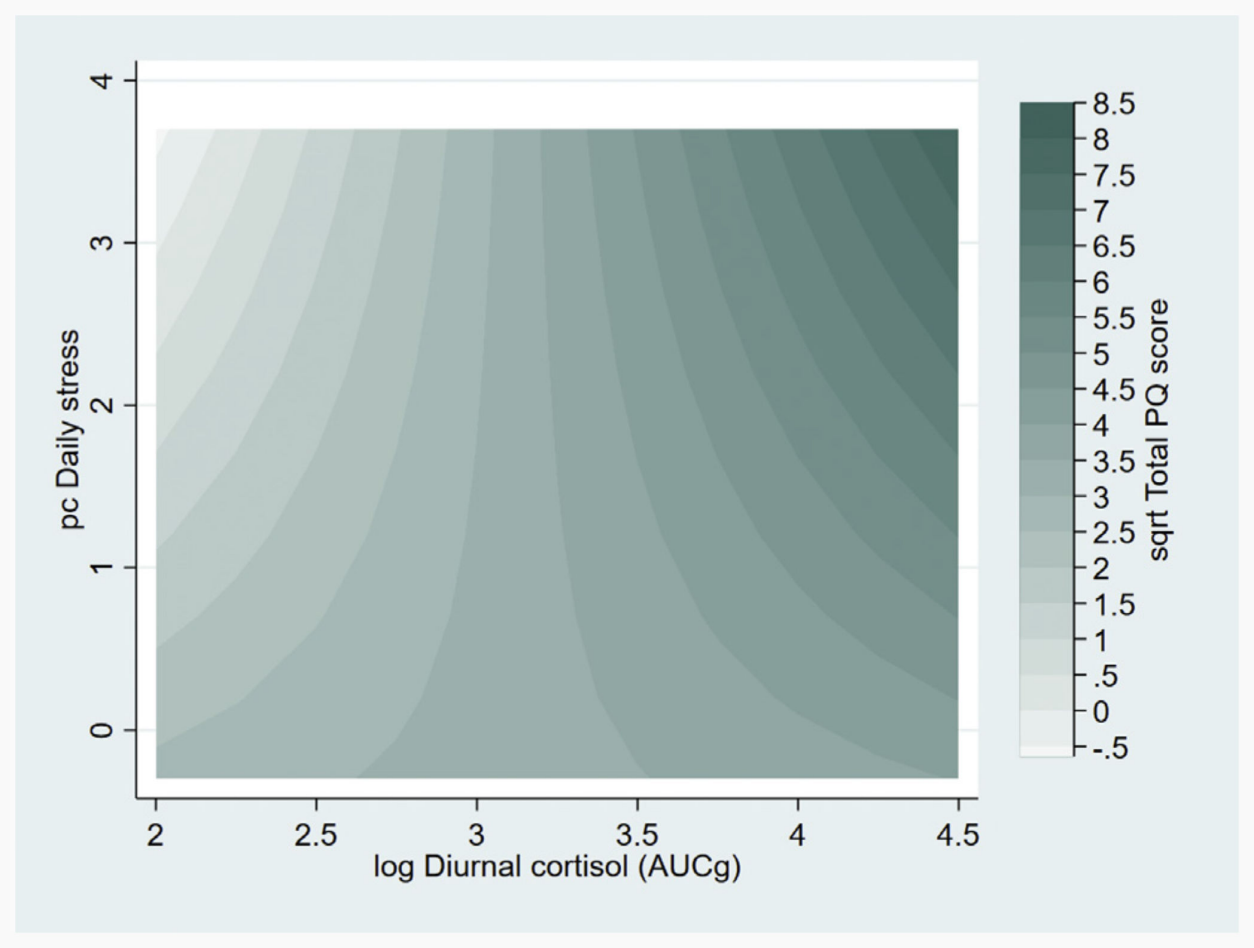
Interaction between diurnal cortisol and daily stressors at time 1 (age 11–14 years) on model-predicted total Prodromal Questionnaire (PQ) score at time 2 (age 17–21 years). Contour plot showing predicted time 2 total PQ scores for varying values of time 1 diurnal cortisol values and daily stressor scores derived from a model including sex, age at time 2, race, family history of schizophrenia/schizoaffective disorder, and time 1 psychopathology as covariates. AUCg, area under the curve with respect to ground; log, log transformed; pc, principal component (score for first pc); sqrt, square root transformed.

Interactions between cortisol and stressor variables were added separately to the base model (Table S12). A significant interaction was observed between diurnal cortisol and daily stressors (β = 0.743, 95% CI: 0.081–1.405), with this model explaining 37% of the variance in T2 total PQ score. As shown in Figure 2, predicted T2 total PQ scores were highest when both diurnal cortisol and daily stressors were increased, lowest when diurnal cortisol levels were low but daily stressor scores were high, and intermediate at median levels of diurnal cortisol irrespective of daily stressor scores.

### Sensitivity Analyses

Analyses conducted with positive scale PQ score as the outcome variable yielded a similar pattern of findings; however, the final model explained a smaller proportion (29%) of the variance (Table S13). Both diurnal cortisol and daily stressors showed only trend-level associations with positive PQ score in the final model (Table S14), and the interaction between these variables failed to reach significance (Table S15). Results for total PQ and positive scale PQ scores were unchanged when analyses were repeated with robust regression (Tables S16–S18), except that the interaction between diurnal cortisol and daily stressors in the model for T2 positive scale PQ score achieved statistical significance (*p* < .05).

## Discussion

To our knowledge, this is the first study to examine the effects of cortisol and stress in late childhood/early adolescence (age 11–14 years) on emerging AP symptoms in young adulthood (age 17–21 years). We found that diurnal cortisol and daily stressors were independently associated with total PQ score after accounting for demographic factors and prior psychopathology and provide preliminary evidence that these factors interacted in predicting total PQ score.

Our finding that adding cortisol and stress measures to the base model increased the proportion of variance explained in total PQ score by 10%, and that diurnal cortisol and daily stressors were independently associated with outcome, is partially consistent with previous studies. In the NAPLS-2 cohort of CHR individuals, childhood trauma and life events were not significant predictors of transition and made little contribution to the multivariable model (38), while basal salivary cortisol significantly improved model performance and was independently associated with transition (35). It is possible that psychosocial stress and basal/diurnal cortisol abnormalities both contribute to the development of AP symptoms but that cortisol plays a greater role in promoting transition to full psychosis among those at CHR. Indeed, psychosocial stress may be so prevalent in the CHR population that it makes little contribution to the shift to psychotic disorder. Our finding that the CAR, unlike diurnal cortisol, was not associated with later AP symptoms is perhaps unsurprising given that studies comparing the CAR in CHR and healthy individuals have yielded inconsistent findings (27,61). Indeed, we previously hypothesized [based on our earlier cross-sectional analyses of data from this cohort (28) and findings from a twin study that investigated heritability of HPA axis markers (62)] that the blunted CAR observed among individuals with psychosis may be genetically mediated (28).

To our knowledge, this is the first study to examine interactions between cortisol and stress in predicting development of AP symptoms, and the significant interaction that we observed between diurnal cortisol and daily stressors (independent of demographic factors and prior psychopathology) is novel. Because these exploratory analyses were not corrected for multiple testing, the results should be interpreted with caution. Given that cortisol and stress measures were collected at the same assessment phase (T1), it is not possible to determine whether cortisol moderates the effect of stress on symptom development or vice versa. The former explanation is, however, consistent with the neural diathesis-stress model (8–10), which proposes that HPA axis changes render individuals with increased vulnerability to psychosis more susceptible to the effects of stress. What drives the initial change in HPA axis function is currently unclear, but it is likely that this reflects a combination of genetic and environmental factors. Indeed, there is evidence to suggest that polymorphisms within the *FKBP5* gene (encoding a protein heavily involved in modulating glucocorticoid receptor function) moderate the effect of childhood trauma on cortisol levels and psychotic symptoms (63,64). Moreover, a recent study observed that an increase in peripheral expression of the *NR3C1* gene (encoding a glucocorticoid receptor) was associated with transition to psychosis among CHR individuals (65). Thus, genetic factors may contribute to HPA axis abnormalities, which in turn increase sensitivity to subsequent stressors that promote psychosis expression (66).

Our sensitivity analyses performed on the PQ positive scale yielded a similar pattern of findings to the main analyses; however, diurnal cortisol and daily stressors failed to achieve statistical significance in the final multivariable model, and the interaction between these variables was also present only at the trend level (except when robust regression was employed). These findings suggest that diurnal cortisol and daily stressors are associated with risk of developing a broad spectrum of AP symptoms (including positive, negative, disorganized, and general symptoms) rather than positive symptoms specifically. This could also explain why previous studies examining associations between cortisol/stress and transition to psychosis in CHR populations (defined as an increase in positive AP symptoms) show inconsistent results. Indeed, examining the effect of cortisol and stress on alternative outcomes (e.g., global functioning) may be a useful direction for further research.

Several limitations should be noted. First, due to the small sample, our analyses (particularly those testing interaction effects) may have been underpowered. Second, we tested 4 interaction effects in separate exploratory models without applying corrections to account for type 1 error. Third, AP symptoms were assessed using a self-report measure rather than interview [which is considered the gold standard (67,68)], which meant that we were unable to examine whether stress and cortisol were associated with fulfillment of CHR criteria. As a related point, using total PQ scores as our primary outcome means that it is difficult to interpret whether the effects of diurnal cortisol and daily stressors are clinically meaningful. However, because assessment of the CHR state is largely confined to specialist early detection/intervention services, whereas screening questionnaires such as the PQ can be easily implemented in nonspecialist teams and general population samples, this strategy increases the translational value of our results.

These limitations notwithstanding, this study emphasizes the importance of conducting longitudinal studies spanning developmental stages. Adolescence is characterized by major changes in the structure, neurochemistry, and connectivity of the brain (69). Our finding that stress and HPA axis markers during late childhood/early adolescence are associated with the emergence of AP symptoms is consistent with the notion that the brain is more sensitive to stressors during this period of neurodevelopmental vulnerability, which may explain why many psychiatric disorders emerge in adolescence (70). In the absence of cortisol data at T2, we are unable to determine whether cortisol levels remained elevated throughout the follow-up periods (i.e., with AP symptoms emerging after a prolonged period of hyperactivity) or whether these cortisol elevations were short-lived but occurred during this critical neurodevelopmental time period. Longitudinal studies incorporating repeated measurements of cortisol are needed to address these questions.

To conclude, we show for the first time that elevated diurnal cortisol and daily stressors in late childhood/early adolescence are independently associated with increased risk for developing AP symptoms in young adulthood and provide preliminary evidence of an interaction between these factors. While these findings require replication in a larger longitudinal study, ideally with repeated measurement of cortisol, they indicate that further research into the predictive utility of salivary cortisol in psychosis high-risk groups is warranted. Our finding that daily stressors, but not negative life events, were associated with development of AP symptoms concurs with previous studies of CHR individuals (32) and suggests that there may be utility in including measures capturing daily stress in predictive models applied to high-risk groups.

## Supplementary Material

Supplementary file

## Figures and Tables

**Table 1:** Sample Characteristics (*N* = 109)

Continuous Variables	Mean	SE
Age at T1, Years	13.21	0.11
Age at T2, Years	17.66	0.08
Lapse of Time Between Assessments, Years	4.45	0.08
BMI at T1, kg/m^2^	19.92	0.34
YSR Internalizing Score at T1	53.13	1.03
YSR Externalizing Score at T1	48.43	0.91
PLEQ-C Score at T1	1.08	0.16
Psychopathology PC Score at T1	−0.04	0.13
Cortisol Awakening Response (AUCi) at T1	89.98	22.67
Diurnal Cortisol (AUCg) at T1	34.36	1.40
Daily Stressor Exposure Score at T1	36.12	1.15
Daily Stressor Distress Score at T1	26.30	1.53
Daily Stressor PC Score at T1	0.01	0.12
Negative Life Event Exposure Score at T1	1.56	0.12
Negative Life Event Distress Score at T1	3.03	0.29
Negative Life Event PC Score at T1	0.01	0.13
Prodromal Questionnaire Total Score at T2	15.79	1.42
Prodromal Questionnaire Positive Scale Score at T2	5.72	0.64
Categorical Variables	*n*	%
Sex, Male	50	45.9
Race		
Black	14	12.8
Other	34	31.2
White	61	56.0
Family History of Sz/SzAff	23	21.1

Missing data: BMI (*n* = 14); YSR internalizing/externalizing (*n* = 5); PLEQ-C (*n* = 4); psychopathology PC (*n* = 5); cortisol awakening response (*n* = 11); diurnal cortisol (*n* = 12); daily stressors exposure/ distress/PC score (*n* = 4); negative life event exposure/distress/PC score (*n* = 4). AUCg, area under the curve with respect to ground; AUCi, area under the curve with respect to increase; BMI, body mass index; PC, principal component; PLEQ-C, Psychotic-Like Experiences Questionnaire for Children; Sz/SzAff, schizophrenia/schizoaffective disorder (at least 1 first- or second-degree relative with confirmed diagnosis); T1, time 1 assessment (age 11–14 years); T2, time 2 assessment (age 17–21 years); YSR, Youth Self-Report.

**Table 2:** Correlations of Demographic Factors and T1 Psychopathology With T1 Predictor Variables and T2 Total and Positive PQ Scores

Demographic Factors	Cortisol Awakening Response	Diurnal Cortisol, Log	Daily Stressor PC Score	Negative Life Event PC Score	PQ Total Score, Sqrt	PQ Positive Scale Score, Sqrt
Age at T1	*r_s_* = 0.185	*r* = −0.010	*r* = 0.062	*r_s_* = −0.024	*r* = −0.019	*r* = −0.015
Age at T2	*r_s_* = 0.191	*r_s_* = −0.045	*r_s_* = 0.205^a^	*r_s_* = −0.019	*r_s_* = 0.029	*r_s_* = −0.030
Time Lapse (T2-T1)	*r_s_* = −0.059	*r* = −0.065	*r* = −0.118	*r_s_* = −0.094	*r* = −0.048	*r* = −0.037
Sex, Male	*r_s_* = −0.207^a^	*r* = −0.110	*r* = −0.018	*r_s_* = 0.096	*r* = −0.237^a^	*r* = −0.109
Family History of Sz/SzAff	*r_s_* = −0.220^a^	*r* = −0.045	*r* = 0.009	*r_s_* = 0.206^a^	*r* =0.123	*r* =0.122
BMI (1/Square) at T1 (kg/m^2^)	*r_s_* = −0.067	*r* = 0.093	*r* = −0.015	*r_s_* = −0.112	*r* = −0.141	*r* = −0.107
Psychopathology at T1	*r_s_* = 0.026	*r_s_* = −0.037	*r_s_* = 0.594^b^	*r_s_* = 0.202^a^	*r_s_* = 0.380^b^	*r_s_* = 0.338^b^

BMI, body mass index; log, log-transformed variable; PC, principal component; PQ, Prodromal Questionnaire; *r*, Pearson’s correlation coefficient; *r_s_*, Spearman’s rho correlation coefficient; sqrt, square root-transformed variable; Sz/SzAff, schizophrenia/schizoaffective disorder; T1, time 1 assessment (age 11–14 years); T2, time 2 assessment (age 17–21 years).

a p < .05.

b p < .001.

**Table 3:** Model Performance Metrics for Prediction of Time 2 Assessment (Age 17–21 Years) Total Prodromal Questionnaire Score

Metrics	Model			
	Base	Base+Cortisol	Base+Stress	Full
*R^2^*	0.23	0.29	0.29	0.35
Adjusted *R^2^*	0.17	0.22	0.22	0.27
*df*	7	9	9	11
*F*	4.14	4.19	4.18	4.26
*p* Value	.001	<.001	<.001	<.001

Base model includes sex, age at time 2 assessment (age 17–21 years), race, family history of schizophrenia/schizoaffective disorder, and time 1 assessment (age 11 –;14 years) psychopathology principal component score as predictors; base + cortisol model additionally includes cortisol awakening response and diurnal cortisol; base + stress model additionally includes daily stressors principal component score and negative life events principal component score; full model includes all variables (full specification of each linear model provided in Supplement). Adjusted *R*^2^, proportion of variance explained by model adjusted for number of predictors; *R*^2^, proportion of variance explained by model; *F*, statistic from *F* test indicating whether model provides better fit than intercept-only model; *p* value, significance for *F* test.

**Table 4:** Multivariable Linear Regression Model for T2 Total Prodromal Questionnaire Scores

Predictor Variables	St. β	β	95% CI	*p* Value
Sex, Male	−0.246	−0.872	−1.555 to −0.188	.013
Age at T2	0.021	0.048	−0.405 to 0.500	.835
Race				
Black	−0.019	−0.118	−1.367 to 1.130	.851
Other	0.160	0.604	−0.131 to 1.339	.106
White	ref	ref	ref	ref
Family History of Sz/SzAff	−0.037	−0.178	−1.150 to 0.795	.717
T1 Psychopathology	0.108	0.148	−0.196 to 0.491	.394
T1 Cortisol Awakening Response (AUCi)	−0.105	−0.001	−0.002 to 0.001	.314
T1 Log Diurnal Cortisol (AUCg)	0.206	0.915	0.062 to 1.769	.036
T1 Daily Stressor PC Score	0.266	0.379	0.034 to 0.723	.032
T1 Negative Life Event PC Score	0.126	0.172	−0.120 to 0.464	.246

Parameters estimated with ordinary least squares regression; total Prodromal Questionnaire scores are square root transformed. AUCg, area under the curve with respect to ground; AUCi, area under the curve with respect to increase; log, log transformed; PC, principal component; ref, reference category; St. β, standardized beta coefficient; Sz/SzAff, schizophrenia/schizoaffective disorder; T1, time 1 assessment (age 11–14 years); T2, time 2 assessment (age 17–21 years).

## Footnotes

1 Measurement of cortisol in blood (serum or plasma) provides information on the total cortisol (i.e., the bound/inactive fraction and unbound/biologically active fraction), whereas only the un-bound fraction passes into saliva (although concentrations are far lower than in serum). Basal cortisol refers to static measures (typically assessed at a single point in time), while diurnal cortisol measures use multiple measures of cortisol collected throughout the day to determine change in secretion (e.g., slope measures) or total cortisol output (e.g., area under the curve measures).

2 The sharp increase in cortisol levels that typically occurs within 15–40 minutes of waking is known as the CAR. The CAR is thought to be a distinct HPA axis component, superimposed on the diurnal profile, that is triggered by the sleep-wake transition and occurs in anticipation of the upcoming demands of the day. Measurement of the CAR requires the acquisition of multiple (typically salivary) cortisol samples during the first hour of the day, commencing at awakening, and is often summarized using area under the curve measures.

## References

[1] Varese F, Smeets F, Drukker M, Lieverse R, Lataster T, Viechtbauer W (2012). Childhood adversities increase the risk of psychosis: A meta-analysis of patient-control, prospective- and cross-sectional cohort studies. Schizophr Bull.

[2] Beards S, Gayer-Anderson C, Borges S, Dewey ME, Fisher HL, Morgan C (2013). Life events and psychosis: A review and meta-analysis. Schizophr Bull.

[3] Myin-Germeys I, Delespaul P, van Os J (2005). Behavioural sensitization to daily life stress in psychosis. Psychol Med.

[4] Myin-Germeys I, van Os J, Schwartz JE, Stone AA, Delespaul PA (2001). Emotional reactivity to daily life stress in psychosis. Arch Gen Psychiatry.

[5] Reininghaus U, Kempton MJ, Valmaggia L, Craig TK, Garety P, Onyejiaka A (2016). Stress sensitivity, aberrant salience, and threat anticipation in early psychosis: An experience sampling study. Schizophr Bull.

[6] Vaessen T, Viechtbauer W, van der Steen Y, Gayer-Anderson C, Kempton MJ, Valmaggia L (2019). Recovery from daily-life stressors in early and chronic psychosis. Schizophr Res.

[7] Martland N, Martland R, Cullen AE, Bhattacharyya S (2020). Are adult stressful life events associated with psychotic relapse? A systematic review of 23 studies. Psychol Med.

[8] Walker EF, Diforio D (1997). Schizophrenia: A neural diathesis-stress model. Psychol Rev.

[9] Pruessner M, Cullen AE, Aas M, Walker EF (2017). The neural diathesis-stress model of schizophrenia revisited: An update on recent findings considering illness stage and neurobiological and methodological complexities. Neurosci Biobehav Rev.

[10] Walker E, Mittal V, Tessner K (2008). Stress and the hypothalamic pituitary adrenal axis in the developmental course of schizophrenia. Annu Rev Clin Psychol.

[11] McCutcheon RA, Krystal JH, Howes OD (2020). Dopamine and glutamate in schizophrenia: Biology, symptoms and treatment. World Psychiatry.

[12] Girshkin L, Matheson SL, Shepherd AM, Green MJ (2014). Morning cortisol levels in schizophrenia and bipolar disorder: A meta-analysis. Psychoneuroendocrinology.

[13] Chaumette B, Kebir O, Mam-Lam-Fook C, Morvan Y, Bourgin J, Godsil BP (2016). Salivary cortisol in early psychosis: New findings and meta-analysis. Psychoneuroendocrinology.

[14] Hubbard DB, Miller BJ (2019). Meta-analysis of blood cortisol levels in individuals with first-episode psychosis. Psychoneuroendocrinology.

[15] Nordholm D, Krogh J, Mondelli V, Dazzan P, Pariante C, Nordentoft M (2013). Pituitary gland volume in patients with schizophrenia, subjects at ultra high-risk of developing psychosis and healthy controls: A systematic review and meta-analysis. Psychoneuroendocrinology.

[16] Ciufolini S, Dazzan P, Kempton MJ, Pariante C, Mondelli V (2014). HPA axis response to social stress is attenuated in schizophrenia but normal in depression: Evidence from a meta-analysis of existing studies. Neurosci Biobehav Rev.

[17] Walker EF, Walder DJ, Reynolds F (2001). Developmental changes in cortisol secretion in normal and at-risk youth. Dev Psychopathol.

[18] Mittal VA, Dhruv S, Tessner KD, Walder DJ, Walker EF (2007). The relations among putative biorisk markers in schizotypal adolescents: Minor physical anomalies, movement abnormalities, and salivary cortisol. Biol Psychiatry.

[19] Mittal VA, Orr JM, Pelletier A, Dean DJ, Smith A, Lunsford-Avery J (2013). Hypothalamic-pituitary-adrenal axis dysfunction in non-clinical psychosis. Psychiatry Res.

[20] Yildirim O, Dogan O, Semiz M, Kilicli F (2011). Serum cortisol and dehydroepiandrosterone-sulfate levels in schizophrenic patients and their first-degree relatives. Psychiatry Clin Neurosci.

[21] Borges S, Gayer-Anderson C, Mondelli V (2013). A systematic review of the activity of the hypothalamic-pituitary-adrenal axis in first episode psychosis. Psychoneuroendocrinology.

[22] Saunders TS, Mondelli V, Cullen AE (2019). Pituitary volume in individuals at elevated risk for psychosis: A systematic review and meta-analysis. Schizophr Res.

[23] Zorn JV, Schür RR, Boks MP, Kahn RS, Joëls M, Vinkers CH (2017). Cortisol stress reactivity across psychiatric disorders: A systematic review and meta-analysis. Psychoneuroendocrinology.

[24] Pruessner M, Béchard-Evans L, Boekestyn L, Iyer SN, Pruessner JC, Malla AK (2013). Attenuated cortisol response to acute psychosocial stress in individuals at ultra-high risk for psychosis. Schizophr Res.

[25] Walter EE, Fernandez F, Snelling M, Barkus E (2018). Stress induced cortisol release and schizotypy. Psychoneuroendocrinology.

[26] Berger M, Kraeuter AK, Romanik D, Malouf P, Amminger GP, Sarnyai Z (2016). Cortisol awakening response in patients with psychosis: Systematic review and meta-analysis. Neurosci Biobehav Rev.

[27] Day FL, Valmaggia LR, Mondelli V, Papadopoulos A, Papadopoulos I, Pariante CM, McGuire P (2014). Blunted cortisol awakening response in people at ultra high risk of developing psychosis. Schizophr Res.

[28] Cullen AE, Zunszain PA, Dickson H, Roberts RE, Fisher HL, Pariante CM, Laurens KR (2014). Cortisol awakening response and diurnal cortisol among children at elevated risk for schizophrenia: Relationship to psychosocial stress and cognition. Psychoneuroendocrinology.

[29] Walker EF, Brennan PA, Esterberg M, Brasfield J, Pearce B, Compton MT (2010). Longitudinal changes in cortisol secretion and conversion to psychosis in at-risk youth. J Abnorm Psychol.

[30] Walker EF, Trotman HD, Pearce BD, Addington J, Cadenhead KS, Cornblatt BA (2013). Cortisol levels and risk for psychosis: Initial findings from the North American prodrome longitudinal study. Biol Psychiatry.

[31] Fusar-Poli P, Borgwardt S, Bechdolf A, Addington J, Riecher-Rössler A, Schultze-Lutter F (2013). The psychosis high-risk state: A comprehensive state-of-the-art review. JAMA Psychiatry.

[32] Thompson KN, Phillips LJ, Komesaroff P, Yuen HP, Wood SJ, Pantelis C (2007). Stress and HPA-axis functioning in young people at ultra high risk for psychosis. J Psychiatr Res.

[33] Labad J, Stojanovic-Pérez A, Montalvo I, Solé M, Cabezas Á, Ortega L (2015). Stress biomarkers as predictors of transition to psychosis in at-risk mental states: Roles for cortisol, prolactin and albumin. J Psychiatr Res.

[34] Valli I, Crossley NA, Day F, Stone J, Tognin S, Mondelli V (2016). HPA-axis function and grey matter volume reductions: Imaging the diathesis-stress model in individuals at ultra-high risk of psychosis. Transl Psychiatry.

[35] Worthington MA, Walker EF, Addington J, Bearden CE, Cadenhead KS, Cornblatt BA (2021). Incorporating cortisol into the NAPLS2 individualized risk calculator for prediction of psychosis. Schizophr Res.

[36] Adam EK, Quinn ME, Tavernier R, McQuillan MT, Dahlke KA, Gilbert KE (2017). Diurnal cortisol slopes and mental and physical health outcomes: A systematic review and meta-analysis. Psychoneuroendocrinology.

[37] Trotman HD, Holtzman CW, Walker EF, Addington JM, Bearden CE, Cadenhead KS (2014). Stress exposure and sensitivity in the clinical high-risk syndrome: Initial findings from the North American Prodrome Longitudinal Study (NAPLS). Schizophr Res.

[38] Cannon TD, Yu C, Addington J, Bearden CE, Cadenhead KS, Cornblatt BA (2016). An individualized risk calculator for research in prodromal psychosis. Am J Psychiatry.

[39] Mason O, Startup M, Halpin S, Schall U, Conrad A, Carr V (2004). Risk factors for transition to first episode psychosis among individuals with ‘at-risk mental states’. Schizophr Res.

[40] Fusar-Poli P, Salazar de Pablo G, Correll CU, Meyer-Lindenberg A, Millan MJ, Borgwardt S (2020). Prevention of psychosis: Advances in detection, prognosis, and intervention. JAMA Psychiatry.

[41] Laurens KR, Cullen AE (2016). Toward earlier identification and preventative intervention in schizophrenia: Evidence from the London Child Health and Development Study. Soc Psychiatry Psychiatr Epidemiol.

[42] Laurens KR, Hodgins S, Maughan B, Murray RM, Rutter ML, Taylor EA (2007). Community screening for psychotic-like experiences and other putative antecedents of schizophrenia in children aged 9–12 years. Schizophr Res.

[43] Laurens KR, Hodgins S, Taylor E, Murray RM, David AS, McGuffin P, Kapur S (2011). Schizophrenia: The Final Frontier.

[44] Maxwell ME (1992). Manual for the FIGS.

[45] Achenbach TM, Rescorla LA (2001). Manual for the ASEBA Preschool Forms & Profiles.

[46] Laurens KR, Hobbs MJ, Sutherland M, Green MJ, Mould GL (2012). Psychotic-like experiences in a community sample of 8,000 children aged 9–11 years: An Item Response Theory analysis. Psychol Med.

[47] Gutteridge TP, Lang CP, Turner AM, Jacobs BW, Laurens KR (2020). Criterion validity of the Psychotic-Like Experiences Questionnaire for Children (PLEQ-C). Schizophr Res.

[48] Mondelli V, Dazzan P, Hepgul N, Di Forti M, Aas M, D’Albenzio A (2010). Abnormal cortisol levels during the day and cortisol awakening response in first-episode psychosis: The role of stress and of anti-psychotic treatment. Schizophr Res.

[49] Belvederi Murri M, Pariante CM, Dazzan P, Hepgul N, Papadopoulos AS, Zunszain P (2012). Hypothalamic-pituitaryadrenal axis and clinical symptoms in first-episode psychosis. Psychoneuroendocrinology.

[50] Nordholm D, Rostrup E, Mondelli V, Randers L, Nielsen MØ, Wulff S (2018). Multiple measures of HPA axis function in ultra high risk and first-episode schizophrenia patients. Psychoneuroendocrinology.

[51] Pruessner JC, Kirschbaum C, Meinlschmid G, Hellhammer DH (2003). Two formulas for computation of the area under the curve represent measures of total hormone concentration versus time-dependent change. Psychoneuroendocrinology.

[52] Cullen AE, Fisher HL, Roberts RE, Pariante CM, Laurens KR (2014). Daily stressors and negative life events in children at elevated risk of developing schizophrenia. Br J Psychiatry.

[53] Heubeck B, O’Sullivan C (1998). An exploration into the nature, frequency and impact of school hassles in the middle school years. Aust Psychol.

[54] Loewy RL, Bearden CE, Johnson JK, Raine A, Cannon TD (2005). The prodromal questionnaire (PQ): Preliminary validation of a self-report screening measure for prodromal and psychotic syndromes. Schizophr Res.

[55] Addington J, Stowkowy J, Weiser M (2015). Screening tools for clinical high risk for psychosis. Early Interv Psychiatry.

[56] Loewy RL, Therman S, Manninen M, Huttunen MO, Cannon TD (2012). Prodromal psychosis screening in adolescent psychiatry clinics. Early Interv Psychiatry.

[57] Rietdijk J, Klaassen R, Ising H, Dragt S, Nieman DH, van de Kamp J (2012). Detection of people at risk of developing a first psychosis: Comparison of two recruitment strategies. Acta Psychiatr Scand.

[58] Gallup JL (2019). Added-variable plots with confidence intervals. The Stata Journal.

[59] Cullen AE, Addington J, Bearden CE, Stone WS, Seidman LJ, Cadenhead KS (2020). Stressor-cortisol concordance among individuals at clinical high-risk for psychosis: Novel findings from the NAPLS cohort. Psychoneuroendocrinology.

[60] Cullen AE, Rai S, Vaghani MS, Mondelli V, McGuire P (2020). Cortisol responses to naturally occurring psychosocial stressors across the psychosis spectrum: A systematic review and meta-analysis. Front Psychiatry.

[61] Pruessner M, Bechard-Evans L, Pira S, Joober R, Collins DL, Pruessner JC, Malla AK (2017). Interplay of hippocampal volume and hypothalamus-pituitary-adrenal axis function as markers of stress vulnerability in men at ultra-high risk for psychosis. Psychol Med.

[62] Wüst S, Federenko I, Hellhammer DH, Kirschbaum C (2000). Genetic factors, perceived chronic stress, and the free cortisol response to awakening. Psychoneuroendocrinology.

[63] Collip D, Myin-Germeys I, Wichers M, Jacobs N, Derom C, Thiery E (2013). FKBP5 as a possible moderator of the psychosisinducing effects of childhood trauma. Br J Psychiatry.

[64] Mondelli V, Ciufolini S (2017). 118.1 effect of the interaction between childhood abuse and FKBP5 gene polymorphism on cortisol awakening response and diurnal cortisol levels in first-episode psychosis. Schizophr Bull.

[65] Iftimovici A, Kebir O, He Q, Jay TM, Rouleau GA, ICAAR Study Group (2020). Stress, cortisol and NR3C1 in at-risk individuals for psychosis: A Mendelian randomization study. Front Psychiatry.

[66] Collip D, Myin-Germeys I, Van Os J (2008). Does the concept of “sensitization” provide a plausible mechanism for the putative link between the environment and schizophrenia?. Schizophr Bull.

[67] Schultze-Lutter F, Renner F, Paruch J, Julkowski D, Klosterkötter J, Ruhrmann S (2014). Self-reported psychotic-like experiences are a poor estimate of clinician-rated attenuated and frank delusions and hallucinations. Psychopathology.

[68] Granö N, Kallionpää S, Karjalainen M, Roine M, Ranta K, Heinimaa M (2016). Discrepancy between self-reported and interviewed psychosis risk symptoms: Auditory distortions are the most reliably reported symptom by self-report. Early Interv Psychiatry.

[69] Paus T, Keshavan M, Giedd JN (2008). Why do many psychiatric disorders emerge during adolescence?. Nat Rev Neurosci.

[70] Gomes FV, Rincón-Cortés M, Grace AA (2016). Adolescence as a period of vulnerability and intervention in schizophrenia: Insights from the MAM model. Neurosci Biobehav Rev.

