# Supplementary material for: Cortisol Levels in Childhood Associated With Emergence of Attenuated Psychotic Symptoms in Early Adulthood
Source: Biol Psychiatry. Author manuscript; Available in PMC 2022 Jun 20. (PMC7612877; doi:10.1016/j.biopsych.2021.08.009)
Supplement: Supplementary file [file EMS145856-supplement-Supplementary_file.pdf]

Cortisol Levels in Childhood Associated with Emergence of Attenuated Psychotic Symptoms in Early Adulthood

Supplemental Information

Participant Recruitment

Participants were drawn from the London Child Health and Development Study (CHADS), for which recruitment has been described in detail previously (1-3). The present study includes children who were recruited to the 'Longitudinal selected sample' (1) who were invited to complete biennial, laboratory-based assessments throughout adolescence. The recruitment strategies for participants in this longitudinal selected sample are described below.

School Screening Procedure

The majority of children in the longitudinal selected sample were recruited using an epidemiologically-informed community screening procedure which involved administering screening questionnaires in school. These questionnaires assessed four risk factors for schizophrenia, as follows. Three of these were well-replicated 'antecedents of schizophrenia' as identified in epidemiological studies, defined as (i) caregiver-reported delays in speech and/or motor development (2), (ii) child-reported Emotional Symptoms and/or caregiver-reported Peer Relationship Problems, Conduct Problems, and/or Hyperactivity-Inattention, as indicated by a score in the clinical range on these psychopathology subscales of the Strength and Difficulties Questionnaire (4), and (iii) the presence of at least one child-reported certainly-true psychotic-like experience as measured with the Psychotic-Like Experiences Questionnaire for Children [PLEQ-C (2, 5)]. The fourth risk factor was a family history of schizophrenia or schizoaffective disorder, as reported by the primary caregiver.

Screening was carried out in 73 government and religious primary schools in Greater London, UK (5). In total, 7966 children aged 9-12 years (representing 95% of eligible children enrolled at participating schools) completed questionnaires independently and anonymously in class, with items read aloud by a researcher (4% of parents and 1% of children refused the child's participation, for reasons unknown). Subsequently, 1504 caregivers (18.9%) completed corresponding questionnaires (matched by code number) at home and returned these via reply-paid mail. Among these, 799 families provided contact information and indicated willingness to be contacted to participate in further research.

Clinical Records Screening

The above method was supplemented with targeted screening of electronic medical records of mental health service users within the South London and Maudsley (SLaM) National Health Service (NHS) Foundation Trust. Medical records were reviewed to identify patients with a diagnosis of schizophrenia or schizoaffective disorder who had a child relative aged 9-12 years. Identified families (n=36) were approached following liaison with the patient's care worker.

Recruitment at Baseline

Of the 835 families (799 + 36) who agreed to be contacted for research, we invited 240 to participate in research assessments at the Institute of Psychiatry, Psychology & Neuroscience, King's College London, UK. These 240 cases were selected because they met inclusion criteria, that is, they presented with (a) none of the four risk factors included in screening questionnaires (recruited to serve as a typically-developing comparison group), (b) one or more of the three antecedents of

schizophrenia (developmental delays, internalising/externalising psychopathology, psychotic-like experiences), or (c) a family history of schizophrenia/schizoaffective [Sz/SzAff] disorder) as indicated in caregiver school screening questionnaires or screening of clinical records and confirmed later by caregiver interview using the Family Interview for Genetic Studies [FIGS (6)]. Children not eligible to participate were those who indicated (either in screening questionnaires, or when further contact was made) insufficient English language ability (child or caregiver) to complete assessments; the presence of a neurological condition in the child that affected milestone attainment or current functioning (e.g., epilepsy or cerebral palsy); a diagnosis of autism, Asperger's or diagnosed learning disability (IQ < 70) in the child; or that the child had previously experienced a psychotic episode or had ever received antipsychotic medication. Whilst there were additional cases within the 835 families who met eligibility criteria, study resources restricted capacity to approach all eligible individuals and other families could not be reached as their contact details had changed. Among the 240 families invited to attend the assessment session, 150 participated.

Sample for the Current Study

Of the 150 children who participated in the initial assessment, 130 were subsequently invited to participate in the longitudinal study involving intensive laboratory-based assessments completed biennially throughout adolescence. The other 20 children were not invited to participate in further assessments due to limited resources to follow-up all of those who initially presented only 2 of the 3 antecedents of schizophrenia, or because a neurological abnormality was identified in MRI scan completed at initial assessment or the child had received treatment for psychiatric disorder at the time of initial assessment. Of these 130 children, 121 completed at least one of the primary exposure measures (cortisol or stress) at the T1 assessment when children were aged 11-14 years. Among these children, 6 presented all four risk factors of interest (i.e., the 3 antecedents and family history of Sz/SzAff), 37 had three risk factors (including 5 with family history of Sz/SzAff), 14 had two risk factors (9 of whom had family history of Sz/SzAff), 9 had a single risk factor (8 of whom had family history of Sz/SzAff), and 56 with no risk factors (though noting that 6 of these children had a family history of bipolar disorder).

Principal Component Analysis

To reduce the number of predictors included in the primary analysis and minimise multicollinearity, principal component analysis (PCA) was applied to T1 psychopathology measures, T1 daily stressor scores, and T1 negative life event scores to yield unrotated components. Results are shown below.

T1 Psychopathology PCA

PCA was applied to the Youth Self-Report [YSR (7)] internalising scale T score, YSR externalising T score, and the Psychotic-Like Experiences Questionnaire for Children [PLEQ-C (2, 5)] total score. Results are shown in Tables S1 and S2.

TABLE S1. Eigenvalues for Psychopathology Scores

Component	Eigenvalue	Proportion	Cumulative
Comp 1	1.963	0.654	0.654
Comp 2	0.579	0.193	0.847
Comp 3	0.458	0.153	1.000

TABLE S2. Principal Components for Psychopathology Scores

Variable	Comp 1	Comp 2	Comp 3
YSR internalising T score	0.598	-0.195	-0.778
YSR externalising T score	0.556	0.800	0.227
PLEQ-C total score	0.578	-0.568	0.586

Applying the Kaiser criterion (8), where only components with Eigenvalues > 1.0 are retained, we retained the first principal component (PC1), which explained 65% of the variance. Spearman's correlation analyses (Table S3) confirmed that all three T1 psychopathology variables showed strong, positive correlations with scores on PC1.

TABLE S3. Correlation Coefficients for Psychopathology PC1

	PC1 score
YSR internalising T score	0.820**
YSR externalising T score	0.798**
PLEQ-C total score	0.749**

Spearman's rho correlations, ** p <0.001

T1 Daily Stressors PCA

PCA was next applied to the daily stressor total exposure score (sum of frequency ratings across 37 items) and total distress score (sum of distress ratings across 37 items), see Tables S4 and S5.

TABLE S4. Eigenvalues for Daily Stressor Scores

Component	Eigenvalue	Proportion	Cumulative
Comp 1	1.718	0.859	0.859
Comp 2	0.282	0.141	1.000

TABLE S5. Principal Components for Daily Stressor Scores

Variable	Comp 1	Comp 2
Daily stressor total exposure score	0.707	0.707
Daily stressor total distress score	0.707	-0.707

PC1, explaining 86% of the variance, was retained. Scores on PC1 showed strong, positive correlations with both the daily stressor total exposure and total distress scores (Table S6).

TABLE S6. Correlation Coefficients for Daily Stressor PC1

	PC1 score
Daily stressor total exposure score	0.927**
Daily stressor total distress score	0.927**

Spearman's rho correlations, ** p <0.001

T1 Negative Life Events PCA

Finally, PCA was applied to the negative life event total exposure score (sum of number of events, maximum 8) and total negative life event distress score (sum of distress ratings across 8 events). Results are shown in Tables S7 and S8.

TABLE S7. Eigenvalues for Negative Life Event Scores

Component	Eigenvalue	Proportion	Cumulative
Comp 1	1.853	0.926	0.926
Comp 2	0.147	0.074	1.000

TABLE S8. Principal Components for Negative Life Event Scores

Variable	Comp 1	Comp 2
Negative life event total exposure score	0.707	0.707
Negative life event total distress score	0.707	-0.707

PC1 explained 93% of the variance and was retained. Scores on this component were strongly correlated with both the negative life event total exposure and total distress scores (Table S9).

TABLE S9. Correlation Coefficients Negative Life Event PC1

	PC1 score
Negative life event total exposure score	0.967**
Negative life event total distress score	0.950**

Spearman's rho correlations, ** p <0.001

Model Specification for Primary Analyses

For the primary analyses, linear regression models were used to examine the effect of the main predictor variables on development of attenuated psychotic symptoms at follow-up. The outcome variable (*y*) for these analyses was the square-root transformed total prodromal questionnaire (PQ) score at T2 (age 17-12 years). The main predictor variables were the cortisol awakening response (CAR), diurnal cortisol, daily stressors, and negative life events at T1.

In order to examine the change in model performance (as determined using the F-test of overall model fit, R^2, and adjusted R^2) after including primary predictor variables, we first derived the 'base model' for total PQ scores which included all covariates of no interest. The latter variables were those found to be significantly associated at the $\alpha = 0.05$ level with any of the primary predictor variables or T2 total PQ score (sex, age at T2, ethnicity, family history of schizophrenia/schizoaffective disorder, T1 psychopathology score). The base model was therefore defined as:

Base model: $y = \beta_0 + \beta_1 Sex + \beta_2 Age\ at\ T2 + \beta_3 Black\ ethnicity + \beta_4 Other\ ethnicity + \beta_5 Family\ history + \beta_6 Psychopathology\ at\ T1$

In the next step, we tested whether inclusion of the CAR (AUCi) and log transformed diurnal cortisol (AUCg) improved prediction of total PQ score over and above the base model. The base + cortisol model was therefore defined as:

Base model + cortisol: $y = \beta_0 + \beta_1 Sex + \beta_2 Age\ at\ T2 + \beta_3 Black\ ethnicity + \beta_4 Other\ ethnicity + \beta_5 Family\ history + \beta_6 Psychopathology\ at\ T1 + \beta_7 CAR + \beta_8 Log\ diurnal\ cortisol$

We then tested whether inclusion of daily stressors and negative life events improved prediction over and above the base model. This model was therefore defined as:

Base model + stress: $y = \beta_0 + \beta_1 Sex + \beta_2 Age\ at\ T2 + \beta_3 Black\ ethnicity + \beta_4 Other\ ethnicity + \beta_5 Family\ history + \beta_6 Psychopathology\ at\ T1 + \beta_7 Daily\ stressors + \beta_8 Negative\ life\ events$

The final model included all covariates of no interest, both cortisol variables and both stress variables, and was therefore defined as:

Full model: $y = \beta_0 + \beta_1 Sex + \beta_2 Age\ at\ T2 + \beta_3 Black\ ethnicity + \beta_4 Other\ ethnicity + \beta_5 Family\ history + \beta_6 Psychopathology\ at\ T1 + \beta_7 CAR + \beta_8 Log\ diurnal\ cortisol + \beta_9 Daily\ stressors + \beta_{10} Negative\ life\ events$

In sensitivity analyses, we performed the same model building steps with the PQ positive scale score (square-root transformed) as the outcome variable (*y*).

Model Specification for Secondary Analyses

For the secondary analyses, we examined interactions between each T1 cortisol variable and each T1 stressor variable in models predicting total PQ score (square-root transformed). Applying the same strategy as above, we tested whether each interaction effect improved model performance over and above the base model. We therefore tested four separate models as follows:

Base model + (CAR × daily stressors): $y = \beta_0 + \beta_1 Sex + \beta_2 Age\ at\ T2 + \beta_3 Black\ ethnicity + \beta_4 Other\ ethnicity + \beta_5 Family\ history + \beta_6 Psychopathology\ at\ T1 + \beta_7 CAR + \beta_8 Daily\ stressors + \beta_9 CAR \times Daily\ stressors$

Base model + (CAR × negative life events): $y = \beta_0 + \beta_1 Sex + \beta_2 Age\ at\ T2 + \beta_3 Black\ ethnicity + \beta_4 Other\ ethnicity + \beta_5 Family\ history + \beta_6 Psychopathology\ at\ T1 + \beta_7 CAR + \beta_8 Negative\ life\ events + \beta_9 CAR \times Negative\ life\ events$

Base model + (diurnal cortisol × daily stressors): $y = \beta_0 + \beta_1 Sex + \beta_2 Age\ at\ T2 + \beta_3 Black\ ethnicity + \beta_4 Other\ ethnicity + \beta_5 Family\ history + \beta_6 Psychopathology\ at\ T1 + \beta_7 Log\ diurnal\ cortisol + \beta_8 Daily\ stressors + \beta_9 Log\ diurnal\ cortisol \times Daily\ stressors$

Base model + (diurnal cortisol × negative life events): $y = \beta_0 + \beta_1 Sex + \beta_2 Age\ at\ T2 + \beta_3 Black\ ethnicity + \beta_4 Other\ ethnicity + \beta_5 Family\ history + \beta_6 Psychopathology\ at\ T1 + \beta_7 Log\ diurnal\ cortisol + \beta_8 Negative\ life\ events + \beta_9 Log\ diurnal\ cortisol \times Negative\ life\ events$

In sensitivity analyses, we repeated the above models with the PQ positive scale score (square-root transformed) as the outcome variable (*y*).

Characteristics of the Current Sample and those Lost to Follow-up

From the longitudinal cohort described above (Supplementary Material, page 1), 121 completed at least one of the primary exposure measures at age 11-14 years (time 1: T1) with 109 successfully followed up at age 17-21 years (time 2: T2). Fisher's exact tests (for categorical variables) and Mann-Whitney U tests (for continuous variables) were performed to compare characteristics of individuals who completed the T2 assessment (current sample: N=109) with those who did not (lost to follow-up after T1: N=12). These analyses showed no significant group differences on any demographic factor, T1 psychopathology variable, or T1 predictor variable (Table S1).

TABLE S10. Comparisons between the Current Sample and those Lost to Follow-up on T1 variables

	Current sample (N=109)		Lost to follow-up (N=12)		P value [b]
Age (years) at T1	13.21	(0.11)	13.28	(0.39)	0.667
Sex (male)	50	(45.9)	9	(75.0)	0.071
Ethnicity, n (%)					0.153
White	61	(56.0)	6	(50.0)	
Black	14	(12.8)	4	(33.3)	
Other	34	(31.2)	2	(16.7)	
Family history of Sz/SzAff [a] n (%)	23	(21.1)	4	(33.3)	0.463
Psychopathology PC score at T1	-0.04	(0.13)	0.34	(0.61)	0.995
Cortisol awakening response (AUCi) at T1	89.98	(22.67)	5.53	(51.18)	0.241
Diurnal cortisol (AUCg) at T1	34.36	(1.40)	37.46	(6.15)	0.713
Daily stressor PC score at T1	0.01	(0.12)	-0.11	(0.53)	0.946
Negative life event PC score at T1	0.01	(0.13)	-0.06	(0.44)	0.694

Mean (SE mean) presented for all continuous variables. [a] At least one first- or second-degree relative with a confirmed diagnosis. [b] All p values are exact owing to sample size. T1: time 1 assessment (age 11-14 years); T2: time 2 assessment (age 17-21 years); Sz/SzAff: schizophrenia/schizoaffective disorder: YSR: Youth Self-Report; PLEQ-C: Psychotic-Like Experiences Questionnaire for Children. PC: principal component (score for first component); AUCi: area under the curve with respect to increase; AUCg: area under the curve with respect to ground. Missing data: psychopathology PC (n=5); Cortisol awakening response (n=11); diurnal cortisol (n=12); daily stressors PC score (n=4); negative life event PC score (n=4).

TABLE S11. Correlations of Cortisol and Stress Measures at T1

	Cortisol awakening response	Log Diurnal cortisol
Daily stressor PC score	r_s = 0.077	r = 0.078
Negative life event PC score	r_s = 0.058	r_s = 0.060

T1: time 1 assessment (age 11-14 years); PC: principal component (score for first component); log: log-transformed.

The lack of association between cortisol and stress measures was expected given that measures were not collected on the same day. A recent study utilising data from the North American Prodrome Longitudinal Study (NAPLS, N=662) reported that associations between basal cortisol and stress measures (daily stressors, life events, and childhood trauma) were only observed when measures were collected on the same day (9). Consistent with these findings, a meta-analysis observed poor concordance between naturally-occurring psychological stressors and cortisol measures in studies examining individuals with established psychosis, at-risk individuals, and healthy controls (10) and noted that this may be explained by the fact that none of the studies collected measures on the same day or accounted for time-lapse between assessment in analyses.

TABLE S12. Linear Regression Models Examining Interaction Effects Between Cortisol and Psychosocial Stress at T1 on Total Prodromal Questionnaire Score T2

T1 Interaction terms	*St.β*	*β*	[95% CI]	*p*	R^2	Adj. R^2
Cortisol awakening response × Daily stressor PC score	0.025	0.000	[-0.001, 0.001]	0.812	0.29	0.21
Cortisol awakening response × Negative life event PC score	0.006	0.000	[-0.001, 0.001]	0.952	0.27	0.19
Diurnal cortisol (log) × Daily stressor PC score	1.855	0.743	[0.081, 1.405]	0.028	0.37	0.30
Diurnal cortisol (log) × Negative life event PC score	1.084	0.407	[-0.316, 1.130]	0.266	0.31	0.24

Parameters estimated with ordinary least-squares regression. Each interaction effect examined in an independent regression model added to base model. Base model includes sex, age at T2, ethnicity, family history of schizophrenia/schizoaffective disorder, and T1 psychopathology. Outcome measure in all models: total prodromal questionnaire scores (sqrt transformed) at T2. T1: time 1 assessment (age 11-14 years); T2: time 2 assessment (age 17-21 years); PC: principal component (score for first component); *St.β*: Standardised beta coefficient; *β*: beta coefficient: CI: confidence interval. R^2: proportion of variance explained by model. Adj. R^2: adjusted R^2 accounting for number of model predictors.

TABLE S13. Model Performance Metrics for Prediction of T2 Positive Scale PQ Score

	Model			
	Base	Base + cortisol	Base + stress	Full
R^2	0.20	0.25	0.24	0.29
Adjusted R^2	0.14	0.17	0.17	0.20
F	3.446	3.331	3.310	3.219
p	0.004	0.002	0.003	0.002

Base model includes sex, age at T2, ethnicity, family history of schizophrenia, and T1 psychopathology PC score as predictors; Base + cortisol model additionally includes cortisol awakening response and diurnal cortisol; Base + stress model additionally includes daily stressors PC score and negative life events PC score; full model includes all variables. R^2: proportion of variance explained by model. Adjusted R^2: proportion of variance explained by model adjusted for number of predictors; F: statistic from F-test indicating whether model provides better fit than intercept-only model; *p*: significance for F-test.

TABLE S14. Multivariable Linear Regression Model for T2 Positive Scale Prodromal Questionnaire Score

	St.β	*β*	[95% CI]	*p*
Male sex	-0.111	-0.286	[-0.806, 0.234]	0.277
Age at T2	-0.022	-0.037	[-0.381, 0.308]	0.833
Ethnicity (white)	[ref]	[ref]	[ref]	[ref]
Black	0.086	0.393	[-0.557, 1.344]	0.412
Other	0.166	0.456	[-0.103, 1.015]	0.109
Family history of Sz/SzAff	0.013	0.046	[-0.694, 0.786]	0.901
Psychopathology PC score	0.145	0.144	[-0.117, 0.406]	0.275
Cortisol awakening response (AUCi)	-0.059	-0.000	[-0.002, 0.001]	0.587
Log Diurnal cortisol (AUCg)	0.194	0.628	[-0.021, 1.277]	0.058
Daily stressor PC score	0.240	0.248	[-0.013, 0.510]	0.063
Negative life event PC score	0.087	0.087	[-0.136, 0.309]	0.440

Parameters estimated with ordinary least-squares regression. T1: time 1 assessment (age 11-14 years); T2: time 2 assessment (age 17-21 years); Sz/SzAff: schizophrenia/schizoaffective disorder: PQ: prodromal questionnaire; sqrt: square-root transformed; log: log transformed; AUCi: area under the curve with respect to increase; AUCg: area under the curve with respect to ground; PC: principal component; *St.β*: standardised beta coefficient; *β:* beta coefficient: CI: confidence interval.

TABLE S15. Linear Regression Models Examining Interaction Effects Between Cortisol and Psychosocial Stress at T1 on Positive Scale Prodromal Questionnaire Score at T2

T1 Interaction terms	*St.β*	*β*	[95% CI]	*p*	R^2	Adj. R^2
Cortisol awakening response × Daily stressor PC score	0.057	0.000	[-0.001, 0.001]	0.600	0.24	0.16
Cortisol awakening response × Negative life event PC score	0.078	0.000	[-0.001,0.001]	0.458	0.23	0.14
Diurnal cortisol (log) × Daily stressor PC score	1.708	0.498	[-0.005, 1.001]	0.052	0.31	0.24
Diurnal cortisol (log) × Negative life event PC score	1.395	0.381	[-0.161, 0.923]	0.166	0.27	0.19

Parameters estimated with ordinary least-squares regression. Each interaction effect examined in an independent regression model added to base model. Base model includes sex, age at T2, ethnicity, family history of schizophrenia/schizoaffective disorder, and T1 psychopathology. Outcome measure in all models: positive scale prodromal questionnaire scores (sqrt transformed) at T2. T1: time 1 assessment (age 11-14 years); T2: time 2 assessment (age 17-21 years); *St.β*: Standardised beta coefficient; *β*: beta coefficient: CI: confidence interval. R^2: proportion of variance explained by model. Adj. R^2: adjusted R^2 accounting for number of model predictors.

TABLE S16. Robust Multivariable Regression Model for T2 Total Prodromal Questionnaire Score

	β	[95% CI]	*p*
Male sex	-0.846	[-1.576, -0.116]	0.024
Age at T2	0.051	[-0.432, 0.535]	0.833
Ethnicity (white)	[ref]	[ref]	[ref]
Black	-0.083	[-1.417, 1.251]	0.902
Other	0.583	[-0.202, 1.369]	0.143
Family history of Sz/SzAff	-0.148	[-1.187, 0.891]	0.778
Psychopathology PC score	0.162	[-0.205, 0.529]	0.381
Cortisol awakening response (AUCi)	-0.001	[-0.003, 0.001]	0.343
Log Diurnal cortisol (AUCg)	0.947	[0.036, 1.859]	0.042
Daily stressor PC score	0.385	[0.017, 0.753]	0.040
Negative life event PC score	0.156	[-0.156, 0.468]	0.322

Parameters estimated with robust regression, no zero weights assigned (Cook's D > 1 for all observations). T1: time 1 assessment (age 11-14 years); T2: time 2 assessment (age 17-21 years); Sz/SzAff: schizophrenia/schizoaffective disorder; PQ: prodromal questionnaire; sqrt: square-root transformed; log: log transformed; PC: principal component (score for first component); AUCi: area under the curve with respect to increase; AUCg: area under the curve with respect to ground; *β:* beta coefficient: CI: confidence interval; ref: reference category.

TABLE S17. Robust Multivariable Regression Model for T2 Positive Scale Prodromal Questionnaire Score

	β	[95% CI]	*p*
Male sex	-0.272	[-0.825, 0.281]	0.331
Age at T2	-0.039	[-0.405, 0.327]	0.833
Ethnicity (white)			
Black	0.452	[-0.559, 1.462]	0.376
Other	0.488	[-0.107, 1.083]	0.106
Family history of Sz/SzAff	0.083	[-0.704, 0.869]	0.835
Psychopathology PC score	0.159	[-0.119, 0.437]	0.258
Cortisol awakening response (AUCi)	0.000	[-0.001, 0.001]	0.810
Log Diurnal cortisol (AUCg)	0.654	[-0.036, 1.345]	0.063
Daily stressor PC score	0.221	[-0.058, 0.500]	0.119
Negative life event PC score	0.074	[-0.162, 0.311]	0.533

Parameters estimated with robust regression, no zero weights assigned (Cook's D > 1 for all observations). T1: time 1 assessment (age 11-14 years); T2: time 2 assessment (age 17-21 years); Sz/SzAff: schizophrenia/schizoaffective disorder; PQ: prodromal questionnaire; sqrt: square-root transformed; log: log transformed; PC: principal component (score for first component); AUCi: area under the curve with respect to increase; AUCg: area under the curve with respect to ground; *β:* beta coefficient: CI: confidence interval.

TABLE S18. Robust Regression Models Examining Interaction Effects Between Cortisol and Psychosocial Stress at T1 on Total and Positive Scale Prodromal Questionnaire Scores at T2

	Total PQ score			Positive scale PQ score		
T1 Interaction terms	*β*	[95% CI]	*p*	*β*	[95% CI]	*p*
Cortisol awakening response × Daily stressor PC score	0.000	[-0.001, 0.001]	0.847	0.000	[-0.001, 0.001]	0.578
Cortisol awakening response × Negative life event PC score	0.000	[-0.001, 0.001]	0.911	0.000	[-0.001, 0.001]	0.536
Diurnal cortisol (log) × Daily stressor PC score	0.794	[0.073, 1.514]	0.031	0.553	[0.031, 1.075]	0.038
Diurnal cortisol (log) × Negative life event PC score	0.456	[-0.318, 1.229]	0.244	0.403	[-0.164, 0.971]	0.161

Parameters estimated with robust regression, no zero weights assigned (Cook's D > 1 for all observations). Each interaction effect examined in an independent regression model added to base model. Base model includes sex, age at T2, ethnicity, family history of schizophrenia/schizoaffective disorder, and T1 psychopathology. Outcome measure in all models: positive scale prodromal questionnaire scores (sqrt transformed) at T2. T1: time 1 assessment (age 11-14 years); T2: time 2 assessment (age 17-21 years); *β*: beta coefficient: CI: confidence interval.

Supplemental References

1. Laurens KR, Cullen AE (2016): Toward earlier identification and preventative intervention in schizophrenia: evidence from the London Child Health and Development Study. *Soc Psychiatry Psychiatr Epidemiol*. 51:475-491.
2. Laurens KR, Hodgins S, Maughan B, Murray RM, Rutter ML, Taylor EA (2007): Community screening for psychotic-like experiences and other putative antecedents of schizophrenia in children aged 9-12 years. *Schizophr Res*. 90:130-146.
3. Laurens KR, Hodgins S, Taylor E, Murray RM (2011): Is earlier intervention for schizophrenia possible?: Identifying antecedents of schizophrenia in children aged 9–12 years. In: David AS, McGuffin P, Kapur S, editors. *Schizophrenia: The Final Frontier*. London: Psychology Press, pp 19-32.
4. Goodman R (2001): Psychometric properties of the strengths and difficulties questionnaire. *J Am Acad Child Adolesc Psychiatry*. 40:1337-1345.
5. Laurens KR, Hobbs MJ, Sutherland M, Green MJ, Mould GL (2012): Psychotic-like experiences in a community sample of 8,000 children aged 9-11 years: An Item Response Theory analysis. *Psychol Med*. 47:1495-1506.
6. Maxwell ME (1992): *Family Interview for Genetic Studies.*: National Institute of Mental Health: St. Louis, MO.
7. Achenbach TM, Rescorla LA (2001): Manual for the ASEBA Preschool Forms & Profiles. Burlington, VT: University of Vermont, Research Center for Children, Youth, & Families.
8. Kaiser HF (1960): The Application of Electronic Computers to Factor Analysis. *Educational and Psychological Measurement*. 20:141-151.
9. Cullen AE, Addington J, Bearden CE, Stone WS, Seidman LJ, Cadenhead KS, et al. (2020): Stressor-Cortisol Concordance Among Individuals at Clinical High-Risk for Psychosis: Novel Findings from the NAPLS Cohort. *Psychoneuroendocrinology*. 115:104649.
10. Cullen AE, Rai S, Vaghani MS, Mondelli V, McGuire P (2020): Cortisol Responses to Naturally Occurring Psychosocial Stressors Across the Psychosis Spectrum: A Systematic Review and Meta-Analysis. *Front Psychiatry*. 11:513.
